# The Relation of Federal to State Quarantine

**Published:** 1897-09

**Authors:** R. M. Swearingen

**Affiliations:** State Health Officer of Texas, Austin, Texas


### TEXAS MEDICAL JOURNAL.

ESTABLISHED JULY, 1885.

PUBLISHED MONTHLY.—SUBSCRIPTION $2.00 A YEAR.

VOL. XIII. AUSTIN, SEPTEMBER, 1897. No. 3.

### Original Contributions.

For the Texas Medical Journal.

### THE RELATION OF FEDERAL TO STATE QUARANTINE.

BY R. M. SWEARINGEN, M. D.,
State Health Officer of Texas, Austin, Texas.

[Read at Conference of State Boards of Health, Nashville, Tenn., August 18, 1897.]

IN 1892, when cholera started out on its pilgrimage of devastation, the governments of all civilized countries inaugurated vigorous measures of protection against its invasion.

As a rule, when men, individually and collectively, do things under the inspiration of a panic, they do very unwise things; and when the danger has passed the folly of their acts becomes apparent.

The national cholera-fright of 1892 and 1893 painfully illustrates this general proposition. The Congress of the United States at that time enacted laws that would not, I verily believe, under ordinary circumstances, receive a moment's consideration. The quarantine act of February 15th, 1893, confers upon the treasurer of the United States government the power to set aside at his option, the health laws and the health officers of any State, and authorizes the president of the United States to formulate rules and regulations in lieu of said laws, and to substitute Federal for State quarantine officers to enforce them; it further imposes upon the treasurer of the United States the duty of making rules for the guidance of State health officers.

Article 3 of the act referred to, directs that "the supervising surgeon-general of the Marine Hospital Service shall, immediately after this act takes effect, examine the quarantine regulations of all the States and municipal boards of health, and shall, under the direction of the secretary of the treasury, co-operate with and aid State and municipal boards of health in the execution and enforcement of the rules and regulations of such boards, and in the execution and enforcement of the rules and regulations made by the secretary of the treasury to prevent the introduction of contagious or infectious diseases into the United States from foreign countries, and from one State or territory into another," etc.; and when said rules and regulations have been made they "shall be promulgated by the secretary of the treasury, and enforced by the sanitary authorities of the States and municipalities, where the State or municipal health authorities will undertake to execute and enforce them." But "if the State or municipal authorities shall fail or refuse to enforce the said rules and regulations, the president shall execute and enforce the same, and shall adopt such measures as in his judgment may be necessary to prevent the introduction and spread of such diseases, and may detail or appoint officers for that purpose."

When this remarkable enactment is stripped of its technical verbiage, in plain English it means that sovereign States can not be entrusted with the police regulations necessary to protect the public health, and that the Federal government, with vastly superior knowledge, must stretch forth its mighty arms for our defense.

In this law there is no provision made for testing the merits of any controversy that might arise between State and Federal authority, nor of deciding questions of competency of any officer, nor for court-martial in case of charges of incompetence or neglect of duty; no civil service examination; no tribunal before which shall be determined the grave question of setting aside the laws of a State. It depends solely upon the opinion of the chief, and his opinion, upon the report of some inspector of the Marine Hospital Service, to the effect that the State rules are not satisfactory. What a parody on constitutional government. When one man, without even the form of trial, can set aside the laws of a State, it is a despotism, subversive of every principle of freedom, and unworthy of the American people.

In order to have unmistakable evidence that the State health officials are complying with all the rules and regulations that have emanated from this great central source of sanitary knowledge, inspectors are sent all along the lines of coast quarantine at stated intervals, and many questions are asked, and the most minute investigation is made into everything pertaining to the administration of State quarantine.

Such surveillance will necessarily lead to complications and embarrassment, no matter how wisely the rules may have been formulated, nor how discreetly they may be executed. An illustration recently occurred in my State. I received the following telegram.

"WASHINGTON, D. C., July 19, 1897.

*Dr. R. M. Swearingen, State Health Officer, Austin Texas:*

"Dr. Magruder, Inspector of the Marine Hospital Service for Texas, reports location at Sabine Pass station dangerous in the extreme. Fifty men working on wharf thirty yards from station; vessels from infected port of Vera Cruz, Mexico, arriving. Can you place inspectors lower down? Immediate action necessary. Please wire. (Signed)

"WYMAN, Surgeon-General."

That telegram had an ominous meaning; a kind of portentious significance, well calculated to disturb the repose of even an old quarantine officer. Fortunately I had, but a few days before the receipt of this startling information, visited the station at Sabine Pass, and I was in a position to know, and did know as much about the situation as did Dr. Magruder, and I can assure this conference that I had not felt a single ripple of apprehension. Dr. A. N. Perkins, the State Quarantine Officer in charge of the station, has served there fifteen years, and is thoroughly familiar with the duties of the position. He is strictly competent, fearless and vigilant. When ships arrive from interdicted places they are carefully inspected, and if any real danger exists the most complete isolation is enforced. When no danger is apprehended he tries, in the interests of commerce, to make the burdens of quarantine as light on sailors and ship owners as can be done consistently with good discipline; but he takes no risks nor chances.

In order to throw the lights on this very dangerous condition of things, it will be necessary to give a little unwritten history. The lesson will not be lost, as it brings out very clearly the advantage of knowing real dangers from imaginary ones, and shows how easy it is for those unacquainted with local interests to be misinformed and misled.

Sabine Pass is a small village at the mouth of the Sabine river, the dividing line between Louisiana and Texas. It has been for a number of years, a lumber shipping point of some importance. Within the last year or two, the channel has been deepened to a depth of twenty-three feet of water, and the trade has greatly increased. The quiet little village has been converted into a bustling town, with limitless possibilities. The Kountz Brothers, of New York, a wealthy firm, have bought up nearly all the river front near the mouth of the river, and are building a new town, to be called by the euphonious name of "Kountzville," in honor of its great founders. The brothers are having cut water slits, or docks, to be leased to private parties or corporations, for exclusive privileges, where railway trains can be run alongside the docks, and discharge or receive freight direct from ships. One of these docks or basins, is excavated within fifty yards of the present State quarantine station, but it was planned at that particular place for the specific purpose of forcing the State to move her station. As the State occupied land claimed by the Kountz Brothers, there was no alternative. But it required time to get permission from the Secretary of War for making permanent improvements on the main channel of a port of entry.

The enterprise was pushed with great energy by the State Health Officer of Texas, but not fast enough for these enterprising town builders. The dock referred to as being near the station, had been rented, I was informed, to a lumber company of Beaumont, for some $500 a month; but the said lumber company will not commence operations until the quarantine station is removed. Of course, five hundred dollars a month is a very important consideration, and the anxiety to secure it made the owners impatient and desirous to have a new station. In fact, all at once they became fearfully apprehensive that a yellow fever epidemic would strike this enterprising community, if the station was not removed instanter. To accelerate my slow movements and hasten the removal of the station to another point, they appealed to the Marine Hospital Service, and an inspector from that department has been assigned to the special duty of watching that port, and the new town has the rare distinction of having her health interests guarded by representatives of both State and national governments.

Let us pause here, and analyze the meaning of the expression "State and Nation," and endeavor to arrive at some conclusion as to the rightful jurisdiction of these respective officers.

Judge Cooley tells us that "A State is a society of men, united together for the purpose of promoting their mutual safety. In American Constitutional Law, the word 'State,' is applied to the several members of the American Union; while the word 'Nation' is applied to the whole body of the people embraced within the jurisdiction of the Federal government. 'Sovereignty,' as applied to States, imports the absolute supreme power by which any State is governed.

"There is a division, however, of the powers of sovereignty between the National and State governments, by *subjects*. The former being possessed of supreme, absolute and uncontrolled power over certain subjects throughout all the States and Territories; while the States have the like complete power within their limits, *over other subjects*. The Constitution is the fundamental law of a State or nation, and it must regulate this division of sovereign powers."

The Declaration of Independence made every State a sovereign and independent State, and individual States at first assumed and performed all the functions of government. It required but a few years to demonstrate the necessity of limitations of authority between States and between the States and the general government. "The Congress of 1775 and 1776 was strictly a revolutionary body, and like all revolutionary bodies its authority was undefined." As the exigencies of occasion evolved the need of changes in the original articles of confederation, the changes have been made; and the friction between the National and State systems of government has been harmoniously adjusted. There has always been a strong party clamoring for greater centralization of power at the national capital, but the old doctrine of State sovereignty that had its birth in the organization of the Federal compact is still adhered to by the majority of the States, and the dignity of Statehood asserted and maintained. Now, jurisdictions are defined, and rights are fixed. Among the exclusive rights thus fixed, and heretofore sedulously guarded, no single right has a firmer hold upon the people than the right to protect their health and lives by local self-governmennt. The officers of the State should be the custodians of the public health of each State, and the assumption on the part of the Federal authorities that a surveillance by their inspectors is necessary, not only violates the Constitution of the United States, but it is tantamount to a declaration that the State officials cannot be entrusted with so important a function.

Judge Cooley in his "Constitutional Limitations" weighed with much ability and deliberation these fine points of governmental policy, and his interpretation is the recognized authority. On page 575 of that work he says: "In the general police power of a State, persons and property are subjected to all kind of restraints and burdens, in order to secure the general comfort, health and prosperity." "In the American constitutional system, the power to establish the ordinary regulations of police has been left with the individual States, and cannot be assumed by the National government." "Neither can the National government, through any of its departments or officers, assume supervision of the police regulations of the States, so long as they do not invade the sphere of national sovereignty, and impede the exercise of any authority which the Constitution has confided to the Nation." "Numerous illustrations," he adds, "might be given of the power of States to make regulations affecting commerce, which is sustainable as regulations of police." "Among these are *quarantine regulations and health laws.*"

Allow me to repeat one paragraph from this great jurist: "Neither can the National government, through any of its departments or officers, assume any supervision of the police regulations of the States." It would be interesting to learn how the expounders of the Constitution can reconcile this clause, prohibiting the supervision of police regulations by the National government over States, with article 3 of the act of 1893, directing the supervising surgeon general immediately after the act takes effect to examine the quarantine laws of all the States, and to do precisely what the Constitution says he shall not do.

Without considering the question of reserved rights and constitutional inhibitions, it seems from the very nature of things that public health could be more satisfactorily protected by the State authorities than by other organizations beyond the State limits.

Officers are responsible to their constituents, and must render an account of their stewardship to them. The endorsements and approvals of the people for all public service is the incentive for faithful performances, and the rewards for duties done. The Surgeon General of the Marine Hospital Service in his office at Washington, cannot feel the responsibility as would the chief health officer of any State; nor could the people of any State have an opportunity to show their approval or disapproval of his official acts.

The only argument brought in support of the proposition to relegate the whole matter of quarantine protection to the general government is that it would be a saving to the State; inasmuch, they say, as a border State keeping up coast quarantine is paying for the protection given the interior States incidentally. Those who advocate this measure on this ground certainly take a superficial view of the subject. An appropriation by the Legislature of a State is more tangible and apparent to the casual observer, but a moment's reflection will satisfy any thoughtful man that one State will not lift the burden of taxation from another State by bearing her quarantine expenses, and that each individual State must, either directly or indirectly, through the tariff system, bear its pro rata share of government cost. Regarding then this question from an ecomomic standpoint, it is fair to assume that the people of a State can select officers as much imbued with patriotic interest, and as fully informed on the subject of economy, as would be representatives of the Federal government.

In conclusion, I beg leave to say that this paper is not written for the purpose of criticising the Marine Hospital Service, but to review the law as enacted in 1893, and to show its defects, That law ought to be repealed, or materially changed. It violates the Constitution of the United States, and insults the dignity of sovereign States, by forcing a surveillance upon them that must be as distasteful to those who execute it as it is humiliating to those who are compelled to submit to it.

I deem it especially incumbent upon this conference to consider and take action upon these great questions. We are confronted by a growing power that threatens to monopolize all sanitary matters and control all systems of public health. The evolution of the Marine Hospital Service within a few years from a charitable institution, caring only for sick sailors, into a vast machine of power, is one of the marvels of the century; and unless a halt is called it foreshadows, at no distant date, the doom of all State and municipal quarantines.

